# Quantification of carbonic anhydrase gene expression in ventricle of hypertrophic and failing human heart

**DOI:** 10.1186/1471-2261-13-2

**Published:** 2013-01-08

**Authors:** Bernardo V Alvarez, Anita L Quon, John Mullen, Joseph R Casey

**Affiliations:** 1 Universidad Nacional de La Plata, La Plata, Argentina; 2 Department of Biochemistry, and Membrane Protein Disease Research Group, University of Alberta, Edmonton, AB, T6G 2H7, Canada; 3 Department of Surgery, University of Alberta, Edmonton, AB, T6G 2H7, Canada

**Keywords:** Heart failure, Carbonic anhydrase, pH regulation, Gene expression, Heart transplant, Cardiac hypertrophy

## Abstract

**Background:**

Carbonic anhydrase enzymes (CA) catalyze the reversible hydration of carbon dioxide to bicarbonate in mammalian cells. Trans-membrane transport of CA-produced bicarbonate contributes significantly to cellular pH regulation. A body of evidence implicates pH-regulatory processes in the hypertrophic growth pathway characteristic of hearts as they fail. In particular, Na^+^/H^+^ exchange (NHE) activation is pro-hypertrophic and CA activity activates NHE. Recently Cardrase (6-ethoxyzolamide), a CA inhibitor, was found to prevent and revert agonist-stimulated cardiac hypertrophy (CH) in cultured cardiomyocytes. Our goal thus was to determine whether hypertrophied human hearts have altered expression of CA isoforms.

**Methods:**

We measured CA expression in hypertrophied human hearts to begin to examine the role of carbonic anhydrase in progression of human heart failure. Ventricular biopsies were obtained from patients undergoing cardiac surgery (CS, n = 14), or heart transplantation (HT, n = 13). CS patients presented mild/moderate concentric left ventricular hypertrophy and normal right ventricles, with preserved ventricular function; ejection fractions were ~60%. Conversely, HT patients with failing hearts presented CH or ventricular dilation accompanied by ventricular dysfunction and EF values of 20%. Non-hypertrophic, non-dilated ventricular samples served as controls.

**Results:**

Expression of atrial and brain natriuretic peptide (ANP and BNP) were markers of CH. Hypertrophic ventricles presented increased expression of CAII, CAIV, ANP, and BNP, mRNA levels, which increased in failing hearts, measured by quantitative real-time PCR. CAII, CAIV, and ANP protein expression also increased approximately two-fold in hypertrophic/dilated ventricles.

**Conclusions:**

These results, combined with *in vitro* data that CA inhibition prevents and reverts CH, suggest that increased carbonic anhydrase expression is a prognostic molecular marker of cardiac hypertrophy.

## Background

Heart failure places an increasingly heavy disease burden on populations world-wide, leading to a need to understand basic mechanisms underlying the disease [1]. Identification of basic mechanisms that promote the downward cascade of heart failure holds the promise to develop targeted new therapeutic strategies. Altered ion homeostasis contributes to hypertrophic heart growth, which impairs the heart’s ability to pump effectively and commonly progresses to heart failure. Cardiac Na^+^/H^+^ exchanger 1 (NHE1) is central to maintenance of intracellular pH. Indeed, experimental and clinical studies demonstrated the pathophysiological implications of increased NHE1 activity during an ischemic episode and in hypertrophy [2-4]. NHE1 activity is detrimental to the myocardium as a result of increased intracellular Na^+^ load, leading to elevated intracellular Ca^2+^ through the action of Na^+^/Ca^2+^ exchanger, NCX1. In some studies, increased cardiac expression of NHE1 protein appears to be involved in the subsequent pathological changes [5]. In human heart failure, however, enhanced NHE1 activity is not correlated with increased NHE1 expression, suggesting a role for activation by post-translational mechanisms [6].

Sustained NHE1 activity requires an acidifying pathway, such as Cl^-^/HCO_3_^-^ exchange mediated by AE3, since NHE activity alkalinizes the cell, resulting in self inactivation through a cytosolic modifier site [7,8]. Hyperactivation of NHE1 and AE3 exchanger are associated with hypertrophic heart growth, in a model of spontaneously hypertensive rats [9]. Maximal activity of AE3 and NHE1 require the catalytic activity of the enzyme carbonic anhydrase (CA), which provides the HCO_3_^-^ and H^+^ substrate for the two transporters [10-12]. Treatment of cultured rat cardiomyocytes with the CA inhibitor, ethoxyzolamide (Cardrase), prevented hormonally-induced hypertrophy and reversed it once established [13]. ETZ also normalized spontaneous Ca^++^ transients induced by pro-hypertrophic hormones, indicating that CA has a role in the elevated Ca^++^ found in the hypertrophic heart [13]. The identity of the CA isoform responsible for the anti-hypertrophic effects was not established in the earlier work. Together, CA inhibition, a therapy previously used for diuresis targeting hypertension and heart failure [14], may be an effective therapeutic approach towards mitigation of the heart disease.

Beneficial effects of NHE inhibition in the failing heart have been suggested on the basis of cellular signalling mechanisms and experimental studies [5,15]. Here we examined the expression of the CA isoforms, CAII and CAIV, in normal, hypertrophic and failing human hearts. Our data lead to the conclusion that increased CA expression is a marker of hypertrophic heart, which progresses towards failure, and suggests that CA inhibition is a point to intervene in the hypertrophic cascade. Limiting substrate availability for NHE1 and AE3 intracellular pH regulatory mechanisms by inhibition of CA will impair the signals that trigger the hypertrophic heart growth.

## Methods

### Human ethics and heart sample collection

The study was approved the Human Research Ethics Board, Faculty of Medicine and Dentistry, University of Alberta and all patients gave written informed consent. To avoid possible disease-specific confounding factors, only samples from patients with early-stage hypertrophy (aortic stenosis, valve replacement or coronary artery by-pass surgery) were used.

End-stage failing hearts (severe cardiomyopathy, heart transplantation), were biopsied following explantation associated with cardiac transplant surgery. Samples were collected from right or left free ventricular wall as indicated. Endomyocardial biopsy samples (EMBs), from patients who were referred to the University of Alberta Hospital for evaluation of cardiomyopathy, were collected by needle biopsy (average diameter 3 mm), and immediately placed into microcentrifuge tubes containing 4°C RNA*later*® (Qiagen, Canada) for storage.

### RNA isolation and cDNA synthesis

RNA was isolated from human heart ventricles and human brain cortex (from Cooperative Human Tissue Network (http://www.chtn.nci.nih.gov/)), and used as a control) with RNeasy Micro kit (Qiagen, Canada), according to the manufacturer’s instructions. Isolated RNA was treated with DNase I (2 U/ng of RNA; Qiagen) at 22°C, 15 min. RNA integrity was confirmed by agarose gel electrophoresis.

cDNA synthesis was carried out with Superscript III reverse transcriptase (Invitrogen, Life Technologies), according to the manufacturer’s instructions. cDNA sequences were obtained from the GenBank sequence database of the National Centre for Biotechnology Information (http://www.ncbi.nlm.nih.gov/). Primers were designed with the Oligo software of the DNA Star program (http://frodo.wi.mit.edu/cgi-bin/primer3/primer3.cgi). In conventional RT-PCR, all primers generated only one amplification band visualized by agarose gel electrophoresis. Sequences for all PCR primers are presented in Additional file 1: Table S1.

### Real-time qPCR

Real-time reverse transcription PCR- Real time PCR was performed with Corbett Rotor-Gene 3000 Real Time analyser (Corbett Life Science, Australia). Real time PCR reaction contained 25 μL with 12.5 μL of 2X Platinum® SYBR® Green qPCR SuperMix-UDG (Invitrogen), 5 pmol/primer and 5 μL of template and cDNA prepared from 100 ng total RNA. Cycle threshold values (Ct) were obtained for CAII, CAIV, CAXIV, ANP, BNP, and GAPDH. GAPDH, assumed not to vary between samples, was used to normalize for differences in the efficiency of mRNA isolation from the samples as follows. Ct values were corrected for each sample by addition or subtraction of cycles so that GAPDH Ct values were the same.

### Protein analysis

Explanted heart samples were rapidly placed in RNA*later*® and stored at 4°C. Tissue was disrupted in PBS Buffer (140 mM NaCl, 3 mM KCl, 6.5 mM Na_2_HPO_4_, 1.5 mM KH_2_PO_4_, pH 7.5), containing protease inhibitors (PI, MiniComplete Tablet, Roche). After disruption, ventricular lysates were prepared by addition of SDS-PAGE sample buffer, heated at 70°C, 3 min, and centrifuged 10 min at 16,110 x g.

### Protein expression and tissue culture

Expression constructs of human CAII, rabbit CAIV, and mouse CAXIV, have been described previously [16-18]. HEK293 cells were individually transfected with empty vector (pCDNA3), CAII, CAIV, or CAXIV, cDNAs [17]. Cells were grown at 37°C in an air/CO_2_ (19:1) environment in DMEM, with 5% (v/v) fetal bovine serum and 5% (v/v) calf serum. Two days post-transfection, HEK293 cells were washed in PBS buffer and cell lysates were prepared by addition of 150 μl SDS-PAGE sample buffer to each 60 mm tissue culture dish. Protein samples were transferred to PVDF membranes and then incubated with rabbit anti-human CAII (rabbit polyclonal H-70, Santa Cruz, CA; 1:1000 dilution), goat anti-CAIV antibody (goat polyclonal N-16; Santa Cruz Biotechnology; 1:500), goat anti-CAXIV antibody (goat polyclonal N19, Santa Cruz Biotechnology; 1:1000), goat anti-atrial natriuretic factor antibody (goat polyclonal N-20; Santa Cruz Biotechnology; 1:200), or rabbit anti-α-actinin antibody (rabbit polyclonal H-300, Santa Cruz, CA, USA; 1:500). Immunoblots were then incubated with donkey anti-rabbit IgG conjugated to horseradish peroxidase (HRP), mouse anti-goat IgG conjugated to HRP, or sheep anti-mouse IgG conjugated to HRP (GE Healthcare, Little Chalfont, UK; 1:2000), as appropriate. Blots were visualized and quantified using ECL reagent and a Kodak Image Station.

### Isolation and culture of cardiomyocytes

Adult mice were anesthetized with sodium pentobarbital, 150 mg/kg i.p. Animal protocols were approved by the University of Alberta Animal Policy and Welfare Committee and performed in accordance with Canadian Council on Animal Care guidelines. Hearts were excised, and ventricular myocytes obtained by enzymatic dissociation [13].

#### Statistics

Data are expressed as mean ± SEM. Student Paired t-test or one-way ANOVA followed by Neuwman-Keuls Multiple Comparison post-test analysis, when appropriate, were used to compare data. P < 0.05 was considered of statistical significance.

## Results

### Patient cohort

Clinical conditions of two patient cohorts are summarized. Patients undergoing cardiac interventions had good prognosis and good cardiac contractility measured by left ventricular ejection fraction values of 59 ± 3% (n = 14, Additional file 2: Table S2). Hearts from patients undergoing cardiac transplantation had poor contractility, left ventricular ejection fraction values of 20 ± 2%, and subjected to cardiac transplantation (n = 13, Additional file 3: Table S3). Patients with prevalence of aortic stenosis (Additional file 2: Table S2), presented heart disease with left ventricular remodeling, including mild to moderate left ventricular concentric hypertrophy, non-hypertrophic right ventricles, and non-left ventricular dysfunction (Stage B of heart failure). Conversely, patients with cardiomyopathies (Additional file 3: Table S3) presented ventricular dilation and severely symptomatic heart failure (Stage D of heart failure). Hearts were classified according to established criteria [19]. Importantly, there was no significant difference in age between the two groups. The overall population with aortic stenosis presented at an average age of 61 ± 4 years, with overrepresentation of males (86%), whereas patients with dilated cardiomyopathies presented at an average age of 51 ± 4 years, and were also overrepresented by males (69%).

### Analysis of gene expression in human heart biopsies

**Figure 1:**
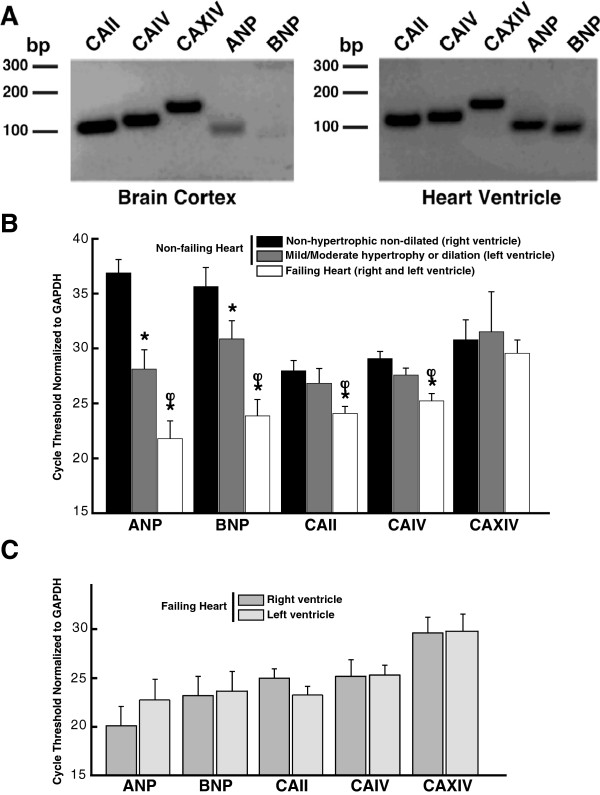
**Carbonic anhydrase mRNA in human hearts.** mRNA, isolated from adult human heart ventricles, or human brain cortex was reverse transcribed, and the resulting cDNA was used as template for PCR (Additional file 1: Table S1). **A**, RT-PCR analysis of the expression levels of mRNA encoding carbonic anhydrase II, IV, and XIV, and encoding hypertrophic markers ANP, and BNP, in adult human ventricle, and adult human brain cortex. PCR products were analyzed on 1% agarose-ethidium bromide gels. **B**, mRNA expression was quantified from ventricles of non-hypertrophic human ventricles (black bars), hypertrophic human ventricles (grey bars), or failing human ventricles (white bars), using real-time quantitative RT-PCR. Data were corrected for individual variation with GAPDH standard curves, expressed as cycle threshold. ^*^P < 0.05 compared to non-hypertrophic ventricle, *Paired T-test*; ^†^P < 0.05 compared to mild/moderate hypertrophic or dilated ventricle, *one-way ANOVA followed by Newman-Keuls Multiple Comparison Test.***C**, cycle threshold values of different genes corrected for individual variation with GAPDH, of right and left failing ventricles.

We examined whether the mRNA and protein expression of carbonic anhydrase genes are altered by cardiac hypertrophy. We analyzed samples derived from single endomyocardial biopsies (EMB), and ventricular slices, respectively, from 27 human samples. An average of 1 mg of total RNA, isolated from each EMB, was subjected to reverse transcription and quantification of transcript abundance by real-time PCR. To verify the specificity of the real-time RT-PCR data, we performed RT-PCR using the designed pair primers (Additional file 1: Table S1) and resolved the products on 1% agarose-ethidium bromide gels. A single band was found for CAII, CAIV, CAXIV, Atrial Natriuretic Peptide (ANP), and Brain Natriuretic Peptide (BNP) transcripts, using either brain cortex, or human heart ventricle RNA (Figure 1A). A weak band was detected for BNP in brain cortex, suggesting low expression in this tissue. The presence of a single band in each case indicates the specificity of amplification, so that quantitative real-time PCR data reports levels of a single species.

Real time reverse transcription PCR quantified the abundance of transcripts in the heart samples. Cycle threshold (Ct) values in real time-PCR were used as a measure of transcript abundance, where higher threshold values correspond to lower mRNA abundance and each change of 1 cycle threshold corresponds to a two-fold difference in message abundance. To correct for differences in total RNA abundance between samples, each sample was analyzed for ANP, BNP and the CA genes, and for GAPDH (Figure 1B). To correct for variations in the amount of mRNA assayed, cycle threshold values were corrected by the Ct for GAPDH, assumed to be present at a constant baseline level.

ANP and BNP are fetal genes whose expression is induced during hypertrophic cardiomyocyte growth, or during heart failure [20,21]. We found marked increases of ANP and BNP expression in all hypertrophic or dilated ventricles (failing heart), compared to non-hypertrophic non-dilated ventricles (non-failing hearts), or compared to ventricles with mild/moderate hypertrophy or dilation (non-failing hearts) (Figure 1B). Remarkably, CAII and CAIV message increased at least two-fold in hypertrophic ventricles, and 16-fold in failing hearts (4 Ct difference), compared to non-hypertrophic, non-dilated non-failing ventricles (Figure 1B). CAXIV mRNA expression did not increase in hypertrophic ventricles of non-failing heart and failing ventricles. We conclude that hypertrophic ventricles express elevated levels of CAII, CAIV, ANP, and BNP, message, relative to non-hypertrophic non-dilated ventricles, which increased in failing ventricles, as evaluated by qRT-PCR. Expression of ANP, BNP, CAII, CAIV, and CAXIV did not differ between right and left failing ventricles (Figure 1C).

### Expression of carbonic anhydrase proteins

To quantify the level of CA protein expression in hearts, immunoblots were performed. Because of the small size of material collected in EMBs, it was not possible to evaluate protein expression in samples from patients with good prognosis (Additional file 2: Table S2). Ventricular samples collected from explanted failing explanted hearts (right or left ventricles), but with no signs of either hypertrophy or dilation, were compared to hypertrophic or dilated failing ventricles (Additional file 3: Table S3).

**Figure 2:**
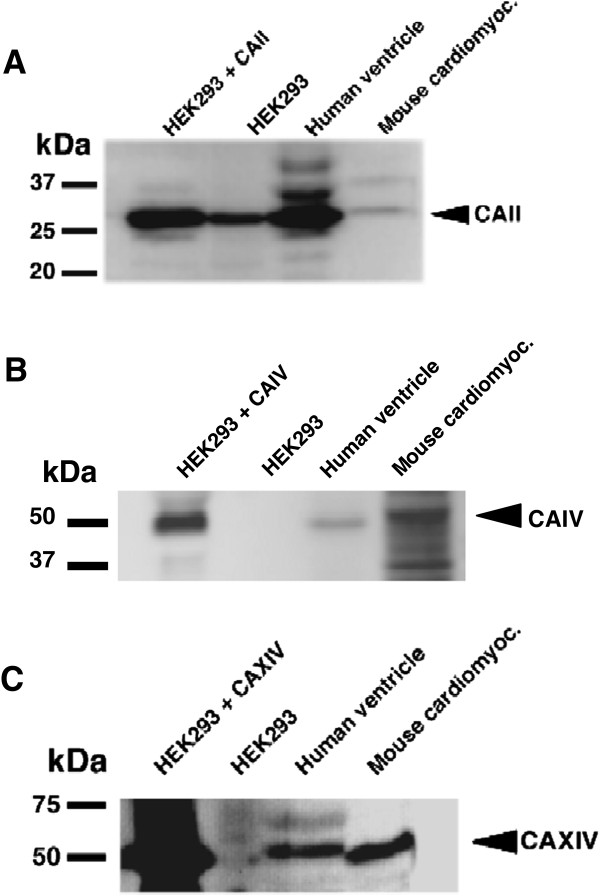
**Expression of carbonic anhydrases in hearts.A**, Lysates were prepared from adult human ventricular sample isolated from explanted heart (see Table two) (50 μg protein), or freshly isolated adult mouse ventricular myocytes (50 μg protein), or HEK293 cells transfected with empty vector (30 μg protein), or HEK293 cells transfected with human CAII (**A**), human CAIV **B**), or human CAXIV (**C**) cDNA (30 μg protein), cDNA. Samples were resolved by SDS-PAGE, transferred to PVDF membrane, and probed with anti-CAII, CAIV or CAXIV as indicated. Filled arrows indicate position of protein.

CAII, a near-ubiquitous cytosolic isoform, is expressed in mouse embryonic and fetal heart, and adult mouse heart [13,22]. We examined the expression of CAII in mouse and human hearts, by immunoblotting. Immunoreactivity was observed in HEK293 transiently transfected with hCAII cDNA, and endogenously in HEK293 cells transfected with empty vector, (Figure 2A). Human heart ventricular samples from explanted hearts, and cardiomyocytes freshly isolated from adult mouse heart, showed robust and moderate CAII expression, respectively (Figure 2A). No immunoreactivity was found on parallel blots incubated with non-immune rabbit serum (not shown). The presence of endogenous CAII in HEK293 cells has been reported previously, using the same antibody [10].

**Figure 3:**
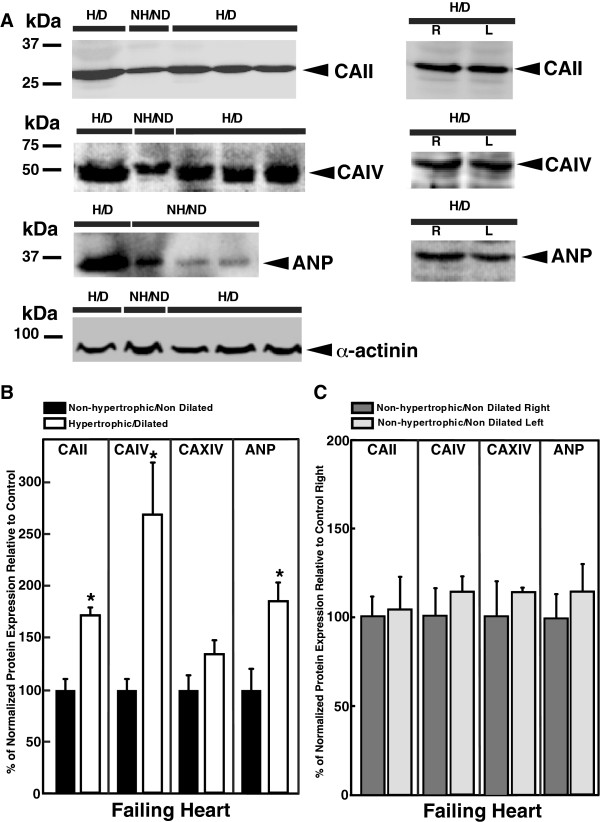
**Expression of CAII, CAIV, and ANP proteins in adult human ventricle.A**, Lysates were prepared from hypertrophic/dilated (H/D), or non-hypertrophic/non-dilated (NH/ND) adult human ventricles, from explanted failing hearts. Left panels, failing ventricles with no signs of either hypertrophy or dilation were compared with hypertrophic or dilated failing ventricles. Protein samples (30 μg) were resolved by SDS-PAGE, transferred to PVDF membrane, and probed with anti-CAII, anti-CAIV, anti-ANP, and anti-α-actinin antibodies. Right panels, samples from left (L) and right (R) ventricles were directly compared. Filled arrow indicates position of protein. **B**, Summary of the protein expression normalized to α-actinin. Values are expressed relative to the non-hypertrophic/non-dilated protein expression; (n = 5). **C**, Summary of the protein expression normalized to α-actinin. Values are expressed relative to the control right ventricle protein expression; (n = 5). *Indicates statistically significant difference (P < 0.05).

To examine the role of CAIV and CAXIV in the failing heart, we studied their expression (Figure 2B,C). Immunoreactive bands, corresponding to the expression of CAIV and CAXIV, were observed in HEK293 cells transiently transfected with rabbit CAIV, or mouse CAXIV (Figure 3A), cDNAs. HEK293 cells transfected with empty vector did not reveal CAIV or CAXIV immunoreactive bands, indicating specificity of the antibodies. Human failing ventricles and isolated adult mouse cardiomyocytes, showed modest and significant CAIV and CAXIV expression, respectively (Figure 2B,C). Specificity of the CAIV antibody is indicated by the lack of band in untransfected HEK293 cells (Figure 2B) and the specificity of the antibody has been previously assessed [23]. Similarly, in untransfected HEK293 cells there is a faint band at the CAXIV position, possibly arising from spillover from the much stronger signal in CAXIV-transfected cells (Figure 2C) and the antibody’s specificity has previously been assessed [18].

Some patients with end-stage heart failure presented with either left or right ventricles with no signs of hypertrophy or dilation (Additional file 3: Table S3). To evaluate whether the CA isoform expression was altered in failing ventricles with hypertrophic or dilated *versus* non-hypertrophic non-dilated ventricles, protein expression was quantified by densitometry of the immunoblots (Figure 3). CAII, CAIV, and ANP proteins could be clearly identified on immunoblots (Figure 3). CAII, CAIV, and ANP protein expression increased ~2 and ~2.5-fold in hypertrophic/dilated ventricles (Figure 3). CAXIV showed a slight increase, ~35%, in dilated failing ventricles compared to non-hypertrophic non-dilated failing ventricles, but did not reach statistical significance difference.

In the failing hearts, CAII, CAIV, and ANP protein expression increased in ventricles without signs of hypertrophy or dilation compared to hypertrophic or dilated ventricles, demonstrating that these genes increased under hypertrophic conditions independent of the contractile performance of the heart. Conversely, failing explanted heart, showed no differences in CAII, CAIV, CAXIV, and ANP protein expression, in non-hypertrophic non-dilated right ventricles, compared to non-hypertrophic non-dilated left ventricles (Figure 3A *right panel* and 3B, n = 3).

## Discussion

This study examined whether altered carbonic anhydrase expression plays a role in human heart failure. Heart failure ranges in severity from moderate impairment in cardiac function, to significant damage that leaves the heart unable to manage its workload [24]. This study demonstrates that CAII and CAIV expression increased in failing human ventricles. These enzymes need to be considered for their contribution to the progression of heart failure and as prognostic markers. A caveat to this work is that the extremely small amount of material in biopsies prevented assessment of changes of carbonic anhydrase catalytic activity.

Similar to our findings, elevated CAII expression was observed in rats with spontaneous hypertension and heart failure (SHHF) [25]. Furthermore, mice that develop angiotensin II-induced cardiac hypertrophy (TG1306/1R, TG), and dilated cardiomyopathy with aging [26], had increased expression of CAII, CAIV, and CAXIV, mRNA, in addition to elevated mRNA for low-activity secreted CAVI [27], suggesting that induction of carbonic anhydrases is a feature of cardiac hypertrophy. Others observed no difference in ANP mRNA abundance between left or right ventricle from control WKY rats, but levels increased in either left or right hypertrophic ventricles of naturally occurring biventricular hypertrophic rats [28].

In the human heart, the right ventricle has important anatomical and functional differences from the left ventricle. The right ventricle is a thin-walled, low-pressure structure that unlike the left ventricle receives most of its blood supply during systole. It has a complex, crescent shape in contrast to the left ventricle with a simple ellipsoid form. Differences between the two ventricles are, however, mostly related to their functions. As the left ventricle must pump blood much further and with more resistance than the right does, the muscular wall of the left ventricle is far thicker to produce the necessary force. Differences in the message and protein for CAs, ANP, and BNP genes expressed in healthy left ventricles compared to healthy right ventricles have not been evaluated here. We were unable to complete this analysis since we could not obtain comparable non-diseased ventricular material.

**Figure 4:**
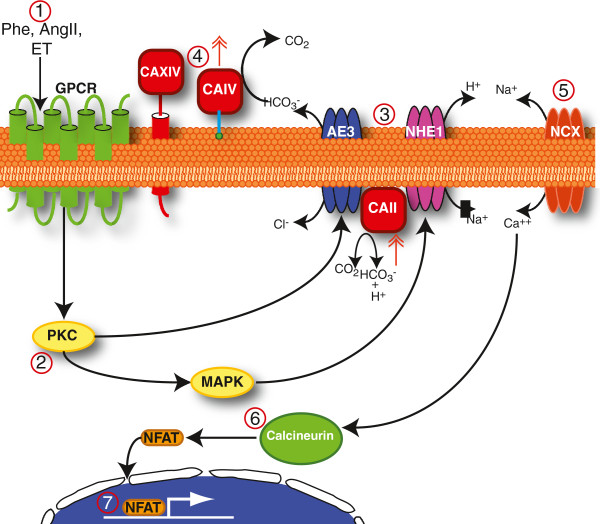
**Integrative model presenting possible pro-hypertrophic action of carbonic anhydrases in cardiomyocytes.** (1) Pro-hypertrophic agonists, including phenylephrine (Phe), Angiotensin II (AngII) and endothelin I (ET) all act upon G-protein coupled receptors (GPCR). (2) In turn protein kinase C (PKC) is activated. (3) PKC directly stimulates AE3 and indirectly stimulates NHE1, via mitogen-activated protein kinase (MAPK). (4) Cl^-^/HCO_3_^-^ exchange and Na^+^/H^+^ exchange by AE3 and NHE1, respectively, are stimulated by the catalytic activity of CAII, CAIV and CAXIV. CAII provides substrate HCO_3_^-^ and H^+^ for efflux respectively by AE3 and NHE1. CAXIV (anchored to the extracellular surface via a transmembrane segment) and CAIV (anchored to the extracellular surface via a glycosylphosphatidyl inositol linkage) consume effluxed HCO_3_^-^ to maximize the size of the transmembrane HCO_3_^-^ gradient, activating AE3. Combined activation of AE3 and NHE1 accumulates Na^+^ cytosolically, without effect on pH [4,9,32,33]. (5) This rise in cytosolic Na^+^ compromises or reverses the ability the Na^+^/Ca^2+^ exchanger (NCX), which normally effluxes Ca^2+^, driven by the transmembrane Na^+^ gradient. (6) Intracellular Ca^++^ thus rises, which activates calcineurin. (7) A major target of calcineurin is the transcription factor, NFAT, which regulates transcription of a set of genes. Here we found that expression of CAIV and CAII in diseased human hearts increased as heart failure progressed (double-headed red arrows).

Carbonic anhydrases work with the AE3 Cl^-^/HCO_3_^-^ exchanger and NHE1 Na^+^/H^+^ exchanger to promote cardiomyocyte hypertrophy, as is found in heart failure (Figure 4). Both AE3 and NHE1 bind to the cytosolic enzyme, CAII, to form a transport metabolon, the complex of a membrane transport protein and the metabolic enzyme responsible for metabolism of the transported substrate [10,11,29]. CAII catalytic activity (CO_2_ + H_2_O↔ HCO_3_^-^ + H^+^) produces HCO_3_^-^ and H^+^ for efflux by AE3, and NHE1, respectively (Figure 4). Combined action of AE3 and NHE1 results in net cellular NaCl loading, without change of pH_i_, which is consistent with the finding of elevated Na^+^ and unchanged pH_i_ in pro-hypertrophically-stimulated cardiomyocytes [30,31]. Co-activation of NHE1-CAII and AE3 is pathological as it is self-sustaining and NHE1 is not subject to inhibition by alkaline pH_i_, since the co-activated transporters do not change pH_i_[4]. We previously found that the activity of AE3fl and NHE1 promote hypertrophy and the hypertrophy-programmed increases expression of the CA enzymes in cultured rat cardiomyocytes [13]. Sustained NHE1/AE3 activation is itself pro-hypertrophic as elevated Na^+^ decreases the efficacy of the Na^+^/Ca^2+^ exchanger, which normally contributes to maintenance of low cytosolic Ca^2+^ levels. In turn, sustained elevated Ca^2+^ is a consummate hypertrophic signal, working through the calcineurin/NFAT signaling cascade.

Recent studies also point to a pro-hypertrophic role of CAII in rodent hearts [34]. Cardiomyocytes Car2 mice [35], which have a disrupted *caii* locus, have decreased cardiomyocyte hypertrophy in response to phenylephrine. Moreover, in rat cardiomyocytes over-expression of a catalytically-null CAII mutant inhibited cardiomyocyte hypertrophy, in a dominant negative manner.

Here we found that CAII and CAIV mRNA levels rise dramatically in hypertrophied and failing human hearts. CAIV has also been found to associate with anion exchangers and to enhance their transport activity, by metabolism of substrate bicarbonate [23]. AE3’s HCO_3_^-^ efflux activity is maximized by CAIV, as conversion of HCO_3_^-^ (by CAIV) maximizes the transmembrane [HCO_3_^-^ gradient, which enhances the rate of HCO_3_^-^ transport. We propose that AE3, and NHE1, CAII, and CAIV work together to promote cardiac hypertrophy (Figure 4). Sustained co-activation of AE3 and NHE1 is pro-hypertrophic, and this is exacerbated by CAII and CAIV, which promote their combined function. The increased expression of CAII and CAIV expression in hypertrophied human myocardium is consistent with a pathological feed-forward cascade in which increased CAII/CAIV expression contributes to hypertrophic signaling, including increased CA expression (Figure 4). To intervene in the hypertrophic cascade present in heart failure we propose that CAII and CAIV represent targets for anti-hypertrophic therapy. Previously, we found that the membrane permeant CA inhibitor, 6-ethoxyzolamide (ETZ, Cardrase), which targets the HTM, intervenes in the feed-forward cascade, preventing and reversing the agonist-induced cardiomyocyte growth [13].

As a counter-point to this argument, however, the role of carbonic anhydrases in moderating the global activity of pH regulatory transporters in heart has been suggested to be modest [36,37]. An alternate explanation for the effects of CAII/CAIV on cardiac hypertrophy could arise through more direct effects on Ca^++^ channels. Alterations of cytosolic pH have profound effects on Ca^++^ channels [38]. Recent findings show that bicarbonate transporters can induce localized changed of cytosolic pH [39], which could be especially significant in confined microenvironments of cardiomyocytes, for example T-tubules. Localized changes of pH, arising from carbonic anhydrase catalysis, could therefore influence Ca^++^ channel activity, with downstream impact on Ca^++^-dependent hypertrophic signaling cascade.

The membrane permeant CA inhibitor, acetazolamide (ACTZ, Diamox), blocks the reabsorption of sodium and potassium by inhibiting CA in the renal tubule [40], and was used as a diuretic in patients with severe congestive heart failure, before the advent of current loop diuretics like furosemide [41]. Clinically, the status of congestive heart failure in all patients receiving Diamox, clearly improved [42]. Patients with mild heart failure were adequately controlled with Diamox, whereas patients with severe heart failure require other diuretics alone, or in combination with Diamox [42]. More recently, ACTZ was safely used in pediatric patients with heart disease, to lower serum bicarbonate and acid–base excess, and raise chloride [43]. High-dose diuretic therapy is the primary cause of metabolic alkalosis in pediatric patients with heart disease, and carbonic anhydrase inhibition improved this condition.

Clinical relevance of the present study includes: 1) Carbonic anhydrase expression levels, in particular CAII and CAIV, increase during progression of cardiac hypertrophy. The prognostic value of biomarkers as clinical predictor factors in heart failure is well-established [44-47] and this work suggests that CAII and CAIV are molecular correlates of hypertrophy. 2) The mainstay of treatment of acute heart failure is diuretic therapy. Diuretics rapidly improve symptoms associated with volume overload, while there are no data showing morbidity or mortality increased from the use of chronic diuretic therapy. CA inhibition with existing drugs could be readily adopted to concomitantly induce diuresis and inhibit the CA enzymes whose activity increases as hypertrophy escalates.

## Conclusion

This study suggests that ventricular hypertrophy/failure and the augmented expression of the CA in the ventricle may be induced by a common mechanism. Ventricular stretch arising from an increased ventricular load leads to the induction of CA gene expression. In this paper we have presented evidence of elevated CA as biomarkers for early detection of cardiac hypertrophy and heart failure, and proposing a mechanism that could improve the cardiac performance, by CA inhibition. The ability to distinguish individual patients at the early stage of heart disease, by detecting elevation of CA expression, might help to improve their functional status and prevent further circulatory collapse that will require cardiac transplantation.

## Abbreviations

AE3: Anion exchanger isoform 3; ACTZ: Acetazolamide; ANP: Atrial natriuretic peptide; BNP: Brain natriuretic peptide; CA: Carbonic anhydrase enzyme; CAII: Carbonic anhydrase isoform II; CAIV: Carbonic anhydrase isoform IV; CAVI: Carbonic anhydrase isoform VI; CAXIV: Carbonic anhydrase isoform XIV; CH: Cardiac hypertrophy; CS: Cardiac surgery; Ct: Cycle threshold; EMB: Endomyocardial biopsy; ETZ: 6-ethoxyzolamide; HT: Heart transplant; HTM: Hypertrophic transport metabolon; HRP: Horse radish peroxidase; NHE: Sodium/proton exchange; PI: Protease inhibitors; qPCR: Quantitative polymerase chain reaction; SHHF: Spontaneous hypertension and heart failure.

## Competing interests

The authors declare that they have no competing interests.

## Authors’ contributions

BV- Manuscript drafting, data acquisition, data analysis. ALQ- data aquisition. JM- data aquisition. JRC- Manuscript drafting, data analysis. All authors read and approved the final manuscript.

## Pre-publication history

The pre-publication history for this paper can be accessed here:

http://www.biomedcentral.com/1471-2261/13/2/prepub

## Supplementary Material

Additional file 1: Table S1 Sequence of primers used in real time polymerase chain reaction. Click here for file

Additional file 2: Table S2 Clinical details of patients undergoing cardiac interventions, who provided endomyocardial biopsy samples. Click here for file

Additional file 3: Table S3 Clinical details of patients undergoing cardiac transplant surgery. Click here for file
