# Supplementary material for: Quantification of carbonic anhydrase gene expression in ventricle of hypertrophic and failing human heart
Source: BMC Cardiovasc Disord. 2013 Jan 8;13:2. doi: 10.1186/1471-2261-13-2 (PMC3570296; doi:10.1186/1471-2261-13-2)
Supplement: Additional file 1: Table S1 — Sequence of primers used in real time polymerase chain reaction. [file 1471-2261-13-2-S1.doc]

**Suppl. Table 1.** Sequence of primers used in real time polymerase chain reaction.

| **Gene/ Human accession number** | Primer Name | **Primer Sequence** | **Position** | **Amplicon size (bp)** |
| --- | --- | --- | --- | --- |
| **Carbonic anhydrase II** NM_000067 | CAII-F | ACTGGGGTTCACTTGATGGA | 351-470 | 119 |
|  | CAII-R | CTGCACAGCTTTCCCAAAAT |  |  |
| **Carbonic anhydrase IV** NM_000717 | CAIV-F | ATACCAGGCCAAACAGTTGC | 369-497 | 128 |
|  | CAIV-R | TCGATGTCCCCTTCTCTTTC |  |  |
| **Carbonic anhydrase XIV** NM_012113 | CAXIV-F | ACAATGCCCAGTCGCCCATC | 433-599 | 166 |
|  | CAXIV-R | CCCAGATACAGGGAGAGGGCAG |  |  |
| **Atrial Natriuretic Peptide** NM_006172 | ANP-F | GCATTCCAGCTCCTAGGTCA | 144-133 | 99 |
|  | ANP-R | TTCCAAATGGTCCAGCAAAT |  |  |
| **Brain Natriuretic Peptide** NP_002521 | BNP-F | CTTGGAAACGTCCGCGTTAC | 212-293 | 81 |
|  | BNP-R | AGGGATGTCTGCTCCACCT |  |  |
| **GAPDH** NM_002046 | GAPDH-F | cagcctcaagatcatcagca | 527-633 | 106 |
|  | GAPDH-R | TGTGGTCATGAGTCCTTCCA |  |  |

GAPDH, glyceraldehyde-3-phosphate dehydrogenase. F and R stand for forward and reverse primers, respectively, in 5’→3’ orientation. Position represents the nucleotide range of the amplicon.
