# Supplementary material for: Quantification of carbonic anhydrase gene expression in ventricle of hypertrophic and failing human heart
Source: BMC Cardiovasc Disord. 2013 Jan 8;13:2. doi: 10.1186/1471-2261-13-2 (PMC3570296; doi:10.1186/1471-2261-13-2)
Supplement: Additional file 2: Table S2 — Clinical details of patients undergoing cardiac interventions, who provided endomyocardial biopsy samples. [file 1471-2261-13-2-S2.doc]

**Suppl. Table 2.** Clinical details of patients undergoing cardiac interventions, who provided endomyocardial biopsy samples.

| Diagnosis/ **Surgical** | **LVH** | **RVH** | **LVEF (%)** |
| --- | --- | --- | --- |
| Aortic stenosis. CABG surgery. | Mild-moderate concentric. | No. | 50-55 |
| Aortic stenosis. CABG surgery; AVR. | Moderate concentric. | No. | 60 |
| Severe mitral valve regurgitation.  CABG surgery. | Mild. Dilation. | No. | 60 |
| Aortic valve stenosis and mitral valve insufficiency. AVR; MVR. | Moderate concentric. Enlargement. | No. | 50-60 |
| Aortic stenosis. Coronary artery disease. CABG surgery; AVR. | Mild concentric. | No. | 50 |
| Aortic stenosis. CABG surgery. | Severe  concentric. | No. | 50-60 |
| Prosthetic valve assessment; AVR. | Moderate concentric. | No. | 50-60 |
| Severe aortic stenosis. AVR; Bentall procedure. | Moderate concentric. | No. | 67 |
| Aortic stenosis; AVR. | Moderate concentric. | No. | 55-60 |
| Severe aortic stenosis. | Moderate concentric. | No. | 67 |
| Severe aortic insufficiency; AVR. | Severe concentric.  Dilated | No. | 86 |
| Severe aortic stenosis; AVR. | Severe concentric. | No. | 50-60 |
| Severe aortic stenosis; AVR. | Mild concentric. | No. | 55 |
| Intraoperative transesophageal echocardiograpy. | Moderate concentric. | No. | 55-60 |

LVH, left ventricular hypertrophy; RVH, right ventricular hypertrophy; LVEF, left ventricular ejection fraction; CABG, coronary artery by-pass grafting; AVR, aortic valve replacement; MVR, mitral valve repair. Among the patients 13/14 were male and average age was 63 years.
