# Supplementary material for: Quantification of carbonic anhydrase gene expression in ventricle of hypertrophic and failing human heart
Source: BMC Cardiovasc Disord. 2013 Jan 8;13:2. doi: 10.1186/1471-2261-13-2 (PMC3570296; doi:10.1186/1471-2261-13-2)
Supplement: Additional file 3: Table S3 — Clinical details of patients undergoing cardiac transplant surgery. [file 1471-2261-13-2-S3.doc]

**Suppl. Table 3**. Clinical details of patients undergoing cardiac transplant surgery.

| **Diagnosis** | **LVH** | **RVH** | **LVEF (%)** |
| --- | --- | --- | --- |
| Dilated cardiomyopathy. | No. Dilated. | No. Dilated. | 24 |
| Viral cardiomyopathy. | No. Dilated. | No. Dilated. | 15 |
| CAD. Status: 2 days past severe myocardial infarction with cardiogenic shock and CABG surgery. | No.  * | No.  * | 20 |
| CAD. Aortic stenosis. Status: patient in very critical state with hemodynamic shock. | Yes. Moderate. | No.  * | 25 |
| Viral myocarditis.  Cardiogenic shock. | No. | No. | - |
| Myocardial infarction with cardiogenic shock. | No.  * | No. Dilated. | 20 |
| CAD. Status: cardiogenic shock. | Yes. Mild-moderate. | No.  * | 25 |
| Biventricular failure secondary to mucopolysaccharidosis. | No.  * | No. Severe dilation. | 34 |
| Cardiogenic shock post MVR/AVR/CABG surgery. | No. Dilated. | No. Dilated. | 20 |
| Viral myocarditis. | No. Severe dilation. | No. Mild dilation. | 10 |
| Severe aortic regurgitation and aortic insufficiency. Congestive heart failure; AVR. | Yes. Moderate eccentric.  Dilation. | No. | 20-25 |
| CAD. Extensive myocardial infarction | No. Severe dilation. | No. Severe dilation. | 17 |
| Viral cardiomyopathy. Severe congestive heart failure. | No. Severe dilation. | No. Mild dilation. | 10 |

LVH, left ventricular hypertrophy; RVH, right ventricular hypertrophy; LVEF, left ventricular ejection fraction; CAD, coronary artery disease; AVR, aortic valve replacement; MVR, mitral valve repair; CABG, coronary artery by-pass grafting.

*****Non-dilated/non-hypertrophic heart ventricles use for immunoblot analysis (Figure 4). Among the patients were nine males and four females. Average age was 51 years.
